# Three-dimensional reconstruction of densely planted rice seedlings based on MultiView images

**DOI:** 10.1016/j.plaphe.2025.100122

**Published:** 2025-09-30

**Authors:** Zhigang Zhang, Liwei Wang, Weiqi Ren, Shoutian Dong, Shaowen Liu, Haoran Xu, Yubo Yang, Rui Gao, Zhongbin Su

**Affiliations:** a Institutions of Electrical and Information, Northeast Agricultural University, Harbin, 150030, China; b Key Laboratory of Northeast Smart Agricultural Technology, Ministry of Agriculture and Rural Affairs, Heilongjiang Province, Harbin, 150030, China

**Keywords:** Multiview image-based 3D reconstruction, Feature extraction, Feature matching, Light stress, Phenotypic parameters

## Abstract

Three-dimensional(3D) seedling reconstruction technology can provide critical technical support for monitoring plant growth, phenotyping high-throughput plants, and conducting precision agriculture. However, multiview image-based reconstruction methods, which rely on image registration and feature matching, are susceptible to issues such as similar textures and viewpoint differences, leading to matching errors and the loss of key structural information. This can result in local deficiencies and reduced accuracy in the reconstructed models. Therefore, to attain improved reconstruction accuracy under low-cost constraints, deep learning-based feature extraction and matching methods are employed in this study, the SuperPoint network is utilized to increase the robustness of the feature point detection and description processes, and the LightGlue algorithm is introduced to improve the accuracy and stability of matching. Additionally, to reduce the impact of shooting and platform jitter on image quality, a dedicated plant 3D reconstruction platform is designed and constructed, and a dataset of densely planted rice seedlings under light stress conditions is collected, comprising three factors (light quality, light quantity, and the photoperiod) ​× ​three levels, totaling nine groups. Experimental results show that the proposed method achieves optimal performance in terms of its point cloud completeness and reprojection error. The phenotypic parameters (e.g., plant height) extracted from the reconstruction data are strongly correlated with the actual measurements (R^2^ ​= ​0.989, RMSE ​= ​4.54 ​mm), validating the potential of the proposed method for applications related to simulating plant growth processes, analyzing the effects of environmental factors (e.g., light), and optimizing crop cultivation schemes.

## 1 Introduction

Rice, as a globally significant food crop, plays a crucial role in ensuring food security, making it highly important to monitor the growth of rice. However, in Northeast China, cold-region rice faces unique challenges because of its specific growth environment, which is characterized by low temperatures and a short growing season. These conditions impose stringent requirements on seedling quality, as the vigor of rice seedlings directly affects their post transplant survival rates, the speed of recovery (regreening), and ultimately, the final yield[1]. Therefore, accurately determining the growth statuses and key phenotypic parameters of cold-region rice seedlings is important for optimizing nursery management processes and improving yields. The traditional plant phenotyping strategy relies primarily on manual observation and hand measurement methods, which are time-consuming, labor-intensive, and inefficient, often resulting in poor data accuracy and consistency. In recent years, three-dimensional reconstruction technology has provided a new approach for conducting plant phenotyping analyses[2]. This technology enables the precise reconstruction of the three-dimensional structures of plants and facilitates the extraction of key geometric parameters such as heights, leaf angles, and volumes[[3], [4], [5]]. Furthermore, on the basis of three-dimensional reconstruction data, it is possible to analyze the effects of different variables (e.g., water, light, and temperature) on seedling growth processes, providing a scientific basis for implementing crop management and environmental regulation[6,7]developed a fully automated segmentation algorithm that is capable of segmenting 3D tomato plant models. Their analysis revealed significant morphological and physiological differences between the early developmental stages observed for different tomato varieties under different water treatments.

Three-dimensional reconstruction technologies can be categorized into two major types: active and passive methods. Active methods rely primarily on external devices and can be further classified into three categories on the basis of their measurement principles: time-of-flight scanners, phase-shift scanners, and active triangulation scanners[8,9]. Light detection and ranging(LiDAR) is one such technology [10].investigated various types of LiDAR systems, including discrete and full-waveform systems, in combination with different assembly configurations for collecting point cloud data. Through a series of algorithms, they were able to extract phenotypic plant traits and quantify multiple phenotypic characteristics, such as height, structure, vegetation density, and coverage.properties [11]developed a high-throughput phenotyping system based on terrestrial LiDAR. By integrating LiDAR and RTK-GPS data, they reconstructed precise three-dimensional models of plants, obtaining phenotypic parameters such as cotton canopy heights, projected canopy areas, and plant volumes. The use of LiDAR for acquiring plant phenotype data has demonstrated significant potential. However, several challenges remain, including the generation of massive amounts of point cloud data via LiDAR, which complicates the data processing and analysis steps. Additionally, LiDAR equipment, particularly high-quality sensors and devices that are capable of capturing fine plant phenotype details, tends to be costly. The sensor of a color-depth camera is an RGB-D sensor, which falls under the category of structured light scanners. A typical consumer-grade example of such a device is the Microsoft Kinect [12]. developed a portable device based on Kinect for measuring the areas of individual leaves in outdoor environments in a noncontact, nondestructive manner [13]. compared the applications of eight depth cameras with different operating principles for performing fruit localization and size measurement in orchards. They established a benchmark root mean square error (RMSE) of 20 ​mm for bias correction when operating at a distance of 2 ​m from the camera to the fruit under sunlight-exposed field conditions. Active 3D reconstruction methods, while offering advantages such as rapid data acquisition processes and real-time performance[14], face limitations because of their complexity, high cost, and susceptibility to the ambient light interference caused by active devices such as laser scanners and structured light systems[15,16]. These constraints make it challenging to satisfy the low-cost and practical requirements for achieving the 3D reconstruction of densely planted rice seedlings. In contrast, passive methods based on computer vision utilize geometric relationships and feature matching between images to reconstruct the three-dimensional structure of a scene. These methods do not require the emission of active light, making them cost-effective approaches[17].

Among the existing passive methods, multiview stereo vision and binocular vision are two common approaches. Binocular vision mimics the working principle of human eyes, utilizing two cameras to capture disparatey information from the same scene to reconstruct its three-dimensional structure[[18], [19], [20]]. Multiview stereo vision involves capturing images from different positions using a camera and leveraging feature point matching and geometric relationships to achieve three-dimensional scene reconstruction[21]. Multiview stereo reconstruction offers advantages such as simple equipment, a low cost, and high flexibility. Considering these factors, this method is the most suitable choice for reconstruction tasks. However, multiview stereo reconstruction relies heavily on image quality and viewpoint variations, making it susceptible to issues caused by lighting changes and regions with sparse textures [22,23], which can result in less accurate 3D models. To address this problem [24],improved their feature extraction algorithm based on the OpenMVG framework. They employed the AKAZE algorithm for performing feature point detection and matching in images, thereby enhancing the detail accuracy of single-plant reconstruction tasks [25]. applied neural radiance field (NeRF) technology during the dense reconstruction phase after conducting sparse 3D reconstruction using COLMAP. By training a neural network to estimate the color and density of each point, they were able to generate high-fidelity 3D plant models. Although the NeRF approach performs well in terms of rendering quality, it differs from reconstruction methods that directly output explicit geometric information (such as discrete point clouds); its output is an implicit volumetric representation (i.e., a radiance field), which requires surface extraction algorithms (such as marching cubes) to generate useable geometric structures. In addition, the authors highlighted certain limitations of their technique, including its slow training speeds and performance constraints due to insufficient sampling [26]. developed a precise pipeline for evaluating maize ear rot based on 3D point cloud technology. They proposed a specialized network called the ear rot segmentation network (ERSegNet) to detect infected regions in maize ears. In their experiments, a "plant-to-camera " mode was adopted for image acquisition purposes, where maize ears were placed at the center of a rotating turntable and spun at a constant speed while a camera captured images from a fixed position, generating 3D point clouds. Although this 3D reconstruction method has demonstrated significant potential for rigid objects such as maize ears[27], for nonrigid objects such as rice seedlings, the leaves may vibrate because of plant movement, introducing significant noise during the 3D model generation step [28]. Therefore, the development of high-precision 3D reconstruction technology that is tailored for nonrigid structures such as rice seedlings is urgently needed. This technology should effectively mitigate dynamic noise, viewpoint variations, and lighting conditions changes, thus providing reliable technical support for monitoring the growth of cold-region rice.

In this study, a novel 3D reconstruction platform based on a "camera-to -rice seedlings" approach is proposed, this method is specifically designed for seedling-stage plants such as rice seedlings, which are characterized by dense planting areas and small sizes. Furthermore, the image feature point extraction and matching process in the 3D reconstruction pipeline is optimized by employing an improved SuperPoint feature extraction method and LightGlue feature matching, significantly enhancing the density and accuracy of the constructed point clouds. This platform enables the generation of high-precision plant point clouds for the dynamic observation of plant growth, and its performance is evaluated in terms of both its reconstruction accuracy and phenotypic parameter extraction precision.

## 2 Materials and methods

### 2.1 Experimental design and data collection

#### 2.1.1 Environmental conditions for plant growth

The experimental data were collected from the stereoscopic rice seedling cultivation facility at Northeast Agricultural University. This study focused on rice seedlings as the research subject, and utilized an artificial climate chamber for environmental control purposes to conduct light stress experiments. In these experiments,pre-soaked rice seeds (Longdao 18) were used, and dry nursery technology was employed for seedling cultivation[29,30]. Prior to the cultivation process, the soil was sun-dried and treated, and nutrient-rich soil was selected as the cultivation substrate to ensure that it satisfied the growth requirements of the rice seedlings before the three-leaf stage. During the cultivation period, in addition to temperature control, light regulation, and regular watering, no additional fertilization was applied. The disinfected and germinated rice seeds were evenly sown at a density of 300 ​g per tray in seedling trays measuring 60 ​cm ​× ​30 ​cm ​× ​2.5 ​cm, with a sowing depth of 1.5 ​cm. After the rice seeds germinated, they were transferred from the dark cultivation chamber for conducting the light stress treatment experiments.

**Fig. 1:**
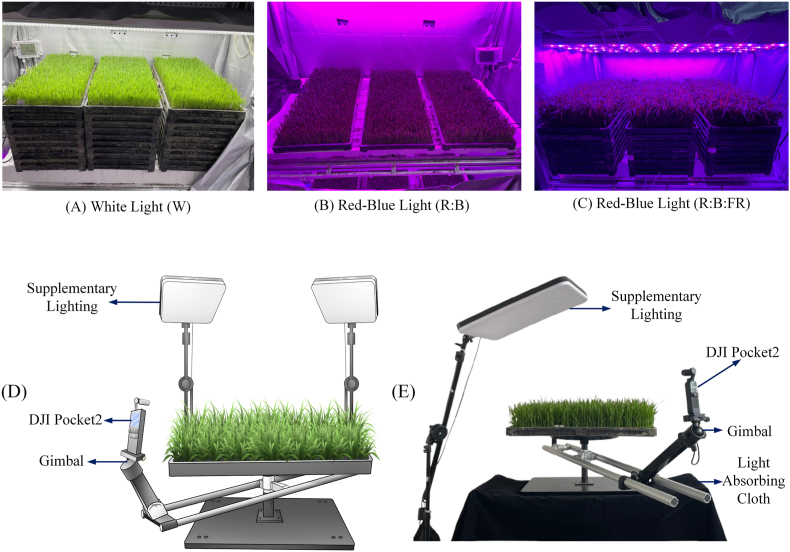
(A) Rice seedlings in the white light cultivation group. (B) Rice seedlings in the red-blue light cultivation group (R: B ​= ​0.7). (C) Rice seedlings in the red-blue-far red light cultivation group (R: B: FR ​= ​62.5:25:12.5). (D) Schematic diagram of the image acquisition platform. (E) Diagram of the image collection platform.

As shown in Fig. 1(A) and (B) and (C), the experiments were conducted in an isolated environment within an artificial climate chamber. The chamber was equipped with Philips LED light sources, with adjustable light intensities ranging from 0 to 1000 ​μmol ​m^−2^ ​s^−1.^ By regulating three key light conditions—the light quality, light quantity (the photosynthetic photon flux density, PPFD), and photoperiod—a dataset of rice seedlings with distinct phenotypic differences was constructed, providing a solid foundational dataset for the subsequent performance validation of the developed 3D reconstruction algorithm. These experiments adopted a three-factor (light quality, light quantity, and photoperiod) ​× ​three-level orthogonal design, which was organized according to the L_9_(3^3^) orthogonal table. A total of nine experimental groups were designed, three replicate seedling trays were established for each treatment group, and identical growth conditions were maintained among the trays within each group to ensure the reliability of the experimental statistics. The three levels of light quality were white light (W), as shown in Fig. 1(A)–a red-to-blue light ratio (R:B ​= ​0.7), as shown in Fig. 1(B)–and a red-blue-far red light combination (R:B:FR ​= ​62.5:25:12.5), as shown in Fig. 1(C). The three light intensity levels were 270 ​μmol ​m^−2^ ​s^−1^ (low light), 340 ​μmol ​m^−2^ ​s^−1^ (medium light), and 510 ​μmol ​m^−2^ ​s^−1^ (high light). The three levels of the photoperiod were: 8 ​h, 12 ​h, and 15 ​h. The experimental treatment combinations were designed using an orthogonal approach. Detailed information on the orthogonal design table is provided in the Supplementary Materials for readers who are interested in the full setup (see Table S1).

To control the interference caused by nonlight stress factors, the environmental conditions were uniformly set as follows: the temperature was dynamically controlled across 16 gradients. Specifically, the period from 0:00 to 5:30 was set as one gradient, the period from 5:30 to 19:30 was divided into 14 gradients, and the period from 19:30 to 0:00 was set as one gradient. The humidity was maintained at a constant 75 ​%.

#### 2.1.2 Data collection and platform construction

In this experiment, two types of data were collected. First, multiview images were captured through a surrounding shooting method to construct a high-precision 3D point cloud model, which served as the data source for the subsequent experiments. Second, during the seedling growth period, phenotypic data were manually collected each day. During the process of collecting the phenotypic data, two of the three seedling trays in each treatment group were designated for manual sampling. Fifty seedlings were randomly selected from each tray, totaling 100 seedlings per treatment group, to calculate the average phenotypic traits. The remaining tray in each group was not subjected to destructive sampling and was reserved for multiview image capture and 3D reconstruction analysis processes. This sampling strategy was consistently applied across all light treatment groups to ensure the comparability and integrity of the data. Data collection began one week after the seedlings germinated. Starting from the one-leaf stage, images of each group of seedlings were taken every three days to obtain data for seedlings under different growth stages and light treatment conditions. To achieve multiview image capture, a “camera-to-plant” data collection platform was designed in this study[31], as shown in Fig. 1(D). In this setup, the seedling tray remained stationary, while the camera rotated around the tray to capture multiview images. The platform consisted of four main components: a rotatable fixed camera arm, a shooting device, a workbench, and a supplementary lighting device. The workbench was made of solid wood with a diameter of 50 ​cm. The camera mounting arm featured a telescopic design, with a gimbal installed at the other end to secure the camera. The fixed arm of the camera could rotate 360° on the workbench, with an adjustable rotation speed enabled to satisfy various shooting requirements. The supplementary lighting device consisted of a 45 ​cm ​× ​32 ​cm full-screen light, with an adjustable color temperature ranging from 2700 ​K to 6500 ​K and a color rendering index (CRI) of Ra 95+.

High-resolution plant images were captured using the DJI Pocket 2 motion camera during the experiment. To reduce motion blur and mitigate image quality issues, the camera was set to operate at a 4 ​K resolution (3840 ​× ​2160 pixels) and a speed of 60 fps. The camera rotated at a speed of 10°/s via a fixed arm to capture 360° omnidirectional images, ensuring that all the details were covered. During the image capture process, the camera collected images at angles of 0°, 30°, and 60° around the plant.

#### 2.1.3 Data processing

After the acquisition procedure, the videos were imported into the FFmpeg video processing software to extract multiview static images. To ensure comprehensive coverage of different perspectives while avoiding data redundancy, the images were captured from the video at a fixed frame rate, with a frame interval of 20 frames, resulting in approximately 330 high-resolution images per seedling tray.

### 2.2 The 3D reconstruction pipeline

**Fig. 2:**
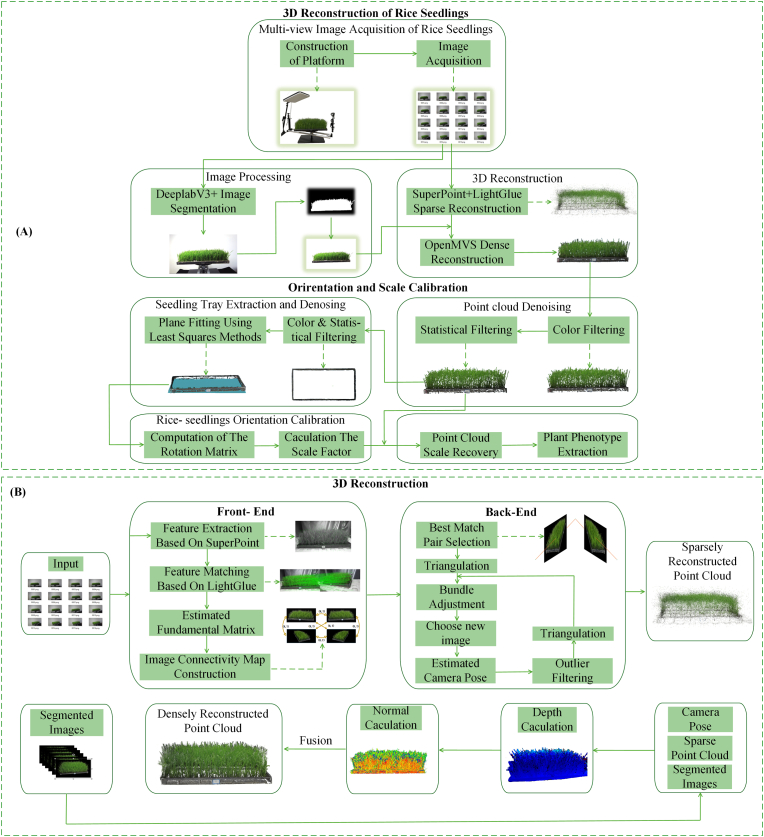
(A)Overall workflow of the 3D reconstruction and phenotypic analysis process for rice seedlings. (B) Detailed workflow of the 3D reconstruction module.

The workflow of the 3D reconstruction and phenotypic extraction method proposed in this study for rice seedlings is illustrated in Fig. 2(A), and the complete technical pathway is as follows. First, 360° omnidirectional high-definition video streams of rice seedlings were captured using a custom-built image acquisition platform. Multiperspective keyframe images were extracted according to the structure-from-motion (SFM) processing requirements. Subsequently, rotation-invariant keypoints and their high-dimensional descriptors were detected through the SuperPoint convolutional neural network, followed by subpixel-level cross-view feature matching, which was implemented via the LightGlue neural network framework. The incremental SFM module in COLMAP was then employed to estimate camera poses and construct a sparse point cloud. For dense reconstruction purposes, the sparse reconstruction results were integrated with the seedling masks generated by the DeepLabV3+ semantic segmentation network. This combined input was processed using the Patch-Match Stereo algorithm of OpenMVS, which performed multiscale pyramidal optimization to achieve dense matching and depth estimation. The initial point cloud underwent denoising through HSV color space filtering and statistical filtering. Finally, the plant point cloud was proportionally calibrated by incorporating prior knowledge of the seedling trays. Orientation calibration was achieved through least-squares plane fitting and rotation matrix computations, ultimately yielding a high-fidelity 3D digital model.

### 2.3 The 3D plant reconstruction based on SuperPoint ​+ ​LightGlue-SFM

#### 2.3.1 SuperPoint feature extraction

In this study, the sparse reconstruction pipeline was optimized and improved on the basis of the COLMAP framework, primarily in terms of two aspects. First, the SuperPoint feature extraction module was introduced to replace the native scale-invariant feature transform (SIFT)-based feature extraction module in COLMAP, enhancing the efficiency and robustness of the feature point detection and description processes. Second, the LightGlue feature matching module was employed to replace the traditional nearest-neighbor matching method, further enhancing the accuracy and stability of feature matching and improving the precision and efficiency of sparse reconstruction in scenarios involving densely planted rice seedling.

**Fig. 3:**
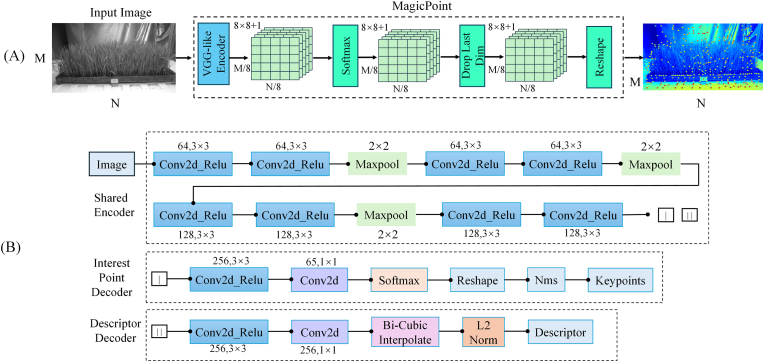
(A) MagicPoint architecture; (B) SuperPoint network architecture.

SuperPoint is a self-supervised keypoint detection and descriptor generation algorithm based on convolutional neural networks[32]. Compared with traditional handcrafted features such as the SIFT and ORB, SuperPoint can automatically learn salient features from images and extract more discriminative and robust descriptors[33,34]. To address the challenge of weak texture‒repetitive texture coupling in the 3D clustered rice seedling reconstruction task, a pretrained SuperPoint network, which was fine-tuned on a custom dataset, was employed in this study. The framework adopts the concept of MagicPoint synthetic data-based pretraining, as illustrated in Fig. 3(A), and exhibits enhanced adaptability to low-texture regions of clustered rice seedlings through the joint optimization of two tasks (feature detection ​+ ​descriptor learning) and a self-supervised fine-tuning mechanism[32]. The SuperPoint architecture, as shown in Fig. 3(B), employs a dual-branch encoder–decoder structure to jointly learn the key point detection and descriptor generation tasks. The input is a single-channel grayscale image, and the model employs a VGG-like encoder structure for feature encoding purposes. The encoder consists of eight 3 ​× ​3 convolutional layers whose channel dimensions are sequentially set to 64, 64, 64, 64, 128, 128, 128, and 128. All the convolutional layers have strides of 1 and are followed by a ReLU activation function. Assuming an input image size of *W* ​× ​*H* ​× ​1, the encoder reduces the dimensionality of the image and outputs a feature map with a size of *W* ​× ​*H* ​× ​128. In the decoding stage, SuperPoint adopts a dual-branch architecture, enabling key point detection and descriptor generation to be simultaneously performed in a single forward pass. For the point-of-interest detection branch, the network generates a key point heatmap, where each pixel value represents the probability of being a feature point. To increase the key point localization accuracy of the model, nonmaximum suppression (NMS) is employed to eliminate redundant detections by preserving only the local maxima within a defined neighborhood while suppressing nonmaximal responses. The descriptor decoder branch refines the feature descriptors through processing steps, including bicubic interpolation, ultimately producing optimized descriptors that precisely characterize the local feature representations around each keypoint. This architecture provides robust feature inputs for the subsequent matching operations.

#### 2.3.2 LightGlue feature matching

In this study, the LightGlue algorithm [35,36] is employed to match the features extracted by SuperPoint. The core of LightGlue is a transformer-based feature matching model, which models the global relationships between keypoints through a multihead cross-attention mechanism, thereby producing more accurate and robust matching results. The formulas for the single-head attention and multihead attention mechanisms are as follows:

(1) $$\begin{array}{c} Attention\left(Q,K,V\right)=softmax\left(\frac{Qk^{T}}{{\mathrm{d}}_{k}}\right)v \end{array}$$

(2) $$\begin{array}{c} M\text{ulti}Head\left(Q,K,V\right)=Concat\left(h\mathrm{e}ad,h\mathrm{e}ad_{2},\ldots ,h\mathrm{e}ad_{h}\right)Wo \end{array}$$

The computation employed for each head is as follows:

(3) $$\begin{array}{c} h\mathrm{e}ad_{i}=attention\left(Qw_{i}^{Q},KW_{i}^{k},VW_{i}^{v}\right) \end{array}$$

**Q:** Query vector, derived from the keypoint features of one image; **K:** key vector, derived from the keypoint features of another image; **V:** value vector, corresponding to the features of the key vector. ${\mathrm{d}}_{k}$**:** dimensionality of the key vector, used for normalization.; $Qw_{i}^{Q},KW_{i}^{k},VW_{i}^{v}$: projection matrices for the query, key, and value, respectively; **W*o***: linear transformation matrix for the output.

The input to LightGlue consists of the SuperPoint features derived from a pair of images, including keypoints and descriptors. First, LightGlue concatenates the 256-dimensional descriptor of each keypoint with its 2D normalized coordinates to form a 258-dimensional feature vector, which is then embedded into the model using sinusoidal positional encoding to preserve the spatial relationships among the keypoints. During the feature selection stage, the model employs an adaptive attention mechanism to filter out the top-K most significant feature points from each image, effectively reducing the computational complexity of the subsequent steps. Next, a multihead cross-attention mechanism is used to compute the attention weights between the two sets of feature points, capturing the global feature relationships between the images. Specifically, the dot product of the query features and key features generates an attention weight matrix, which is normalized by the softmax function and then weighted and summed with the value features to produce updated feature representations. To capture multilevel feature relationships, this process is performed in parallel across multiple attention heads. Additionally, LightGlue introduces a confidence prediction module that combines feature similarity and local geometric consistency to predict the reliability score of each match. This is based on the following formula:

(4) $$\begin{array}{c} c\left(k_{a},k_{b}\right)=\sigma \left(\alpha \cdot sim\left(f_{a},f_{b}\right)+\beta \cdot geo\left(k_{a},k_{b}\right)\right) \end{array}$$

(5) $$\begin{array}{c} M=\left\{\left(k_{a},k_{b}\right)|c\left(k_{a},k_{b}\right)>\tau \right\} \end{array}$$

$f_{a},f_{b}\text{:}$ Descriptors of the matched points $k_{a},k_{b}$; $sim\left(f_{a},f_{b}\right)$**:**descriptor similarity; $geo\left(k_{a},k_{b}\right)$**:**geometric consistency score of the matched points; α**,** β:weight coefficients of the two scores. M*:*a set of keypoint pairs that are determined to correctly correspond between the two images through the algorithm.

Finally, only matched point pairs with confidence scores above a preset threshold of 0.3 are retained, effectively filtering out mismatches. The entire matching process is iteratively optimized through multiple rounds, significantly improving the accuracy and reliability of matching. Compared with the traditional nearest-neighbor matching methods, LightGlue has notable performance advantages in scenarios with varying lighting conditions and complex textures. Even in cases where highly similar feature points and matching difficulties arise because of the presence of repetitive textures at the edges of densely planted rice leaves, LightGlue is still capable of obtaining high-quality matches. This provides high-quality inputs for the subsequent point cloud generation and camera pose estimation processes in 3D modeling.

#### 2.3.3 Plant image segmentation based on DeepLabV3+

In images captured using the “camera-to-rice seedlings” mode, the background typically consists of fixed structures such as walls and platforms. While these elements are irrelevant to the target in 3D reconstruction tasks, they may have varying effects on different stages of the reconstruction process. When SFM is employed for sparse reconstruction purposes, images without plant segmentation contain more feature points because of the presence of structured backgrounds such as scaffolds or platforms with regular geometric patterns. This approach enhances the efficiency of feature matching and the accuracy of image registration relative to those achieved using images with plant segmentation[37]. However, during the dense reconstruction stage, the inclusion of background information leads to the generation of many irrelevant points, increasing the noise density of the point cloud and reducing the reconstruction accuracy attained for the plant region. To improve the overall quality of 3D reconstruction, the accurate extraction of the foreground regions of plants and the effective removal of background interference are necessary. Therefore, in this study, a two-stage reconstruction strategy is proposed. During the SFM stage, the original images are used for feature extraction and matching to increase the success rate of feature matching between images and avoid registration failures caused by insufficient feature points; during the dense reconstruction stage, background-free images generated by DeepLabV3+ are used as inputs to minimize the interference of background noise during the generation of dense point clouds.

Through atrous convolution and an atrous spatial pyramid pooling (ASPP) module, the DeepLabV3+ model can accurately capture the target plant regions at multiple scales and segment the foreground plant regions from complex backgrounds[38]. Utilizing the image collection method described in Section 2.1.3, a dataset is created by selecting 600 images of rice plants at different growth stages and manually annotating them using LabelMe. The dataset is randomly divided into training, validation, and test sets at a 6:2:2 ratio. The DeepLabV3+ network is trained to perform segmentation, and the trained segmentation model is subsequently used to obtain mask images for dense 3D reconstruction. By performing pixel-level operations between the semantic segmentation masks and the original images, RGB images containing only the foreground regions are obtained. The annotation results produced for the rice seedlings are shown in Fig. S1(A) in the Supplementary Materials.

#### 2.3.4 The 3D rice seedling reconstruction

The 3D reconstruction of rice seedlings is primarily divided into two stages: sparse reconstruction and dense reconstruction. The detailed reconstruction workflow is illustrated in Fig. 2(B). In the sparse reconstruction stage, SuperPoint is first employed to extract key feature points from the original images; this is followed by feature matching using LightGlue to obtain highly robust correspondences. Subsequently, leveraging the pose estimation module in COLMAP, the rotation and translation parameters (i.e., extrinsic parameters) of the cameras are estimated on the basis of the geometric relationships of the matched points. Through pose estimation, the spatial geometric relationships between images can be recovered, accurately determining the relative positions and orientations of each image. Next, bundle adjustment is applied to minimize the image reprojection error, and all the camera poses and 3D point positions are jointly optimized. Finally, on the basis of the optimized camera poses and spatial points, a sparse 3D point cloud model is generated. In the dense reconstruction stage, background-removed images generated by the DeepLabV3+ model are used as inputs, and the point clouds and camera poses derived from the sparse reconstruction procedure are fed into the OpenMVS framework. First, the depth map computation module of OpenMVS generates depth maps for each image using multiview geometric relationships; then, unreliable depth values are filtered out through pixel consistency checks and depth optimization; finally, the depth maps acquired from all viewpoints are fused to produce high-resolution dense point clouds.

### 2.4 Point cloud denoising and scale restoration

#### 2.4.1 Comprehensive plant denoising

After the 3D rice plant reconstruction process is completed, the point cloud data often contain noise and outliers, primarily because of various factors in the reconstruction pipeline. First, although the foreground regions of the plants are extracted through semantic segmentation prior to beginning reconstruction, some background pixels may still be misclassified as foreground pixels, causing the background point cloud to “adhere” to the plant point cloud and form edge noise. Second, during the data acquisition procedure, owing to leaf occlusion and feature matching errors, outliers and geometric inaccuracies are inevitably present in the 3D point cloud. Additionally, interference and reflections from supplemental lighting in the artificial climate chamber introduce noise at the edges of the plants.

(1) Color Filtering Based on the HSV Color Space

Given that edge noise primarily originates from background regions and lighting reflections and that the color characteristics of these noise points significantly differ from those of the plant regions, a color filtering method based on the HSV (hue, saturation, value) color space is employed in this study to eliminate specific types of noise points[39]. The specific workflow is as follows. First, the RGB color values of each point in the point cloud are converted to the HSV color space. The HSV color space effectively separates the hue, saturation, and brightness features of colors, facilitating a more accurate target region point screening procedure. On the basis of the results of the experimental analysis and the color characteristics of the rice plants, the HSV parameter ranges are defined. Next, each point in the point cloud is traversed, and its HSV values are extracted and compared with the predefined threshold ranges. By constructing a color classification function, the point cloud is divided into foreground points (conforming to the plant color range) and noise points (background points). The noise points are marked and removed using condition-based filtering, thus generating optimized point cloud data. Finally, the HSV parameter ranges are iteratively adjusted on the basis of the effectiveness of edge noise removal after performing color filtering. By analyzing the distribution and quantity of the edge noise in the filtered point cloud, the threshold settings are progressively optimized until the noise removal effect is maximized.

(2) Outlier Detection and Removal Based on Statistical Methods

To remove edge noise and outliers from the plant point cloud, a statistical-based outlier detection and removal method is employed in this study. First, outlier removal is based on calculating the average distance of the point set within the neighborhood of each point. For each discrete point ρ, the KD-tree fast retrieval method is used to establish a neighborhood point set $N_{p}=\left\{q_{1},\ldots ,q_{m}\right\}$, centered on the sampling point, where *m* is the predefined number of nearest neighbor search points. Afterward, the average neighborhood distance $\bar{d_{P}}$ and the standard deviation of the dispersion $\sigma_{p}$ are calculated for each point.

(6) $$\begin{array}{c} \bar{d_{p}}=\frac{1}{m}\sum_{j=1}^{m}{\mathrm{d}}_{p,qj}=\frac{1}{m}\sum_{j=1}^{m}{\left\|p-q_{j}\right\|}_{2} \end{array}$$

(7) $$\begin{array}{c} \sigma_{p}=\sqrt{\frac{1}{m}\sum_{j=1}^{m}{\left(d_{P,qj}-\bar{d_{P}}\right)}^{2}} \end{array}$$

Furthermore, by analyzing the average distance and standard deviation metrics of all the points in $N_{P}$, an overall average distance $\bar{D_{Np}}$ and a standard deviation of dispersion $\Sigma_{Np}$ can be obtained, reflecting the local distribution characteristics.

(8) $$\begin{array}{c} \bar{D_{Np}}=\frac{1}{m}\sum_{j=1}^{m}\bar{d_{qj}} \end{array}$$

(9) $$\begin{array}{c} \Sigma_{Np}=\frac{1}{m}\sum_{j=1}^{m}\sigma_{qj} \end{array}$$

The overall point cloud of the rice plants satisfies a Gaussian distribution, which is characterized by an average distance value and a standard deviation. If the average distance of a point significantly exceeds the global average $\bar{D_{Np}}$ and standard deviation $\Sigma_{Np}$, that point can be identified as an outlier and removed.

#### 2.4.2 Plant size calibration based on prior information

The initial size of the plant point cloud model is relative and is determined by the intrinsic and extrinsic parameters of the camera as well as the utilized 3D reconstruction algorithm; however, it does not directly correspond to the actual physical dimensions. To ensure that the physical size of the rice plant point cloud model aligns with the real-world scene, a calibration method based on the geometric information of the seedling tray is proposed in this study. The point cloud is proportionally corrected using the actual dimensions of the seedling tray.

(1) Extraction of the Seedling Tray Region

The extraction of the seedling tray region from the point cloud is based on a color filtering method. Leveraging the significant color differences between the tray (black), rice seedlings (green), and soil (dark brown), the HSV color space is used to define the color ranges, and the PCL library is employed to isolate the point cloud of the seedling tray region. A statistical-based outlier detection method is subsequently applied to remove the noise points, further enhancing the quality of the point cloud data.

(2) Orientation Calibration

The orientation of the seedling tray in the point cloud coordinate system is irregular and requires calibration to align its geometric orientation with the standard coordinate system. First, the least-squares method is used to fit the plane of the seedling tray in the point cloud, and a principal component analysis (PCA) is applied to extract its normal vector. The deviation between this normal vector and the standard Z-axis (0,0,1) is calculated by using Rodrigues' rotation formula to determine the rotation matrix $R_{z}$. Next, horizontal alignment is performed by projecting the point cloud of the seedling tray region onto the XY-plane. The convex hull algorithm is used to extract the boundary points of the projected point cloud, and PCA is applied to the extracted boundary point set. The angle between the direction vector V1 of the long side of the tray and the standard X-axis direction X=(1, 0, 0) is calculated as follows:

(10) $$\begin{array}{c} \theta =arc\;\cos\frac{v_{1}\cdot x}{\left\|v_{1}\right\|\left\|x\right\|} \end{array}$$

A rotation matrix is then constructed around the Z-axis:

(11) $$\begin{array}{c} R_{z}=\left[\begin{array}{ccc} \cos\;\theta & -\sin\;\theta & 0\\ \sin\;\theta & \cos\;\theta & 0\\ 0 & 0 & 1 \end{array}\right] \end{array}$$

The point cloud is rotated to align the long side of the seedling tray with the X-axis.

(3) Plant Scale Restoration

In the rotated coordinate system, $x_{\min},x_{m_{ax}}=\min\left(p_{x}\right),\max\left(p_{x}\right)$; $y_{\min,}y_{\max}$ ​= ​$\min\left(p_{y}\right),\max\left(p_{y}\right)$,and $z_{\min,}z_{\max}$ ​= ​$\min\left(p_{z}\right),\max\left(p_{z}\right)$

The dimensions of the seedling tray are: $L_{cloud}$ ​= ​$x_{m_{ax}}-x_{\min}$;$W_{cloud}=\max\left(p_{y}\right)-\min\left(p_{y}\right)$,and $H_{cloud}$ ​= ​$\max\left(p_{z}\right)-\min\left(p_{z}\right)$.

By using the known actual physical dimensions of the seedling tray, ($L_{real}=60cm,W_{real}=30cm,H_{r}=2.5cm$)

The scale factor of the point cloud is calculated as follows: $s_{L}=\frac{L_{real}}{L_{cloud}}$, $s_{w}=\frac{W_{real}}{W_{cloud}}$, $s_{H}=\frac{H_{real}}{H_{cloud}}$

If the ratio is known, the average scale factor is taken. Finally, a scale transformation is applied to all the coordinates of the point cloud:

(12) $$\begin{array}{c} p_{scale}=S\cdot P_{rotated} \end{array}$$

After scaling, the physical dimensions of the point cloud align with the actual physical scene.

### 2.5 Joint evaluation method

To comprehensively evaluate the accuracy of the 3D reconstruction and population phenotypic trait analysis method proposed in this study, a systematic analysis and discussion are conducted on the proposed method. First, the performance of the feature point extraction scheme is assessed by statistically analyzing the number of feature points and their distribution uniformity to validate the effectiveness of the feature extraction mechanism. Second, the feature point matching results are evaluated, with a focus on the matching accuracy rate to reflect the robustness of the matching process. With respect to the image segmentation results, metrics such as the intersection over union (IoU), precision, and recall are subsequently used to assess the accuracy of the model in terms of segmenting the foreground of the rice plants. Next, by comparing the completeness and performance of the rice point cloud models generated by the different methods, the 3D reconstruction accuracy improvement achieved by the proposed method is verified. Finally, by combining population phenotype data (e.g., plant heights), the error between the extracted values and manually measured values is calculated, and the root mean square error (RMSE) is used as an evaluation metric to validate the reliability of the point cloud model for phenotypic data extraction purposes.

(13) $$\begin{array}{c} RMSE=\sqrt{\frac{\sum_{i=1}^{N}{\left(H_{p}^{i}-H_{actual}^{i}\right)}^{2}}{N}} \end{array}$$

(14) $$\begin{array}{c} R^{2}=1-\frac{\sum_{i=1}^{N}{\left(H_{actual}^{i}-H_{P}^{i}\right)}^{2}}{\Sigma_{\mathrm{i}=1}^{N}{\left(H_{actual}^{i}-\bar{H_{actual}}\right)}^{2}} \end{array}$$

$H_{p}^{i}$: The point cloud-measured average plant height for the i-th seedling tray in the population; $H_{actual}^{i}$: the actual manually measured average plant height for the i-th seedling tray. ${\bar{H}}_{predicted}$: The mean plant height value derived from point cloud measurements; ${\bar{H}}_{actual}$: the mean plant height value derived from manual measurements; *N*: the number of samples.

## 3 Results

### 3.1 Experimental environment

The experiments are conducted on a Ubuntu 20.04 system equipped with an Intel Core i9-9900 ​K CPU, an NVIDIA RTX 3090 GPU, and 24 ​GB of available memory. The implementation utilizes Python and C++ with PyTorch as the deep learning framework. Visual Studio serves as the integrated development environment. During 3D reconstruction, COLMAP is used for sparse reconstruction, and OpenMVS is used for dense reconstruction, while point cloud processing is performed using the point cloud library (PCL).

### 3.2 SuperPoint & LightGlue evaluations

#### 3.2.1 SuperPoint feature extraction evaluation

To systematically evaluate the feature extraction performance of SuperPoint in 3D plant reconstruction tasks, the SIFT is used in this study as the baseline method, and three other feature extraction methods are compared: ORB, SIFT, and SuperPoint. The experiments are conducted using a multiview image dataset for the rice plants. The evaluation focuses on three performance metrics: the number of feature points, feature point quality, and feature extraction time.

**Fig. 4:**
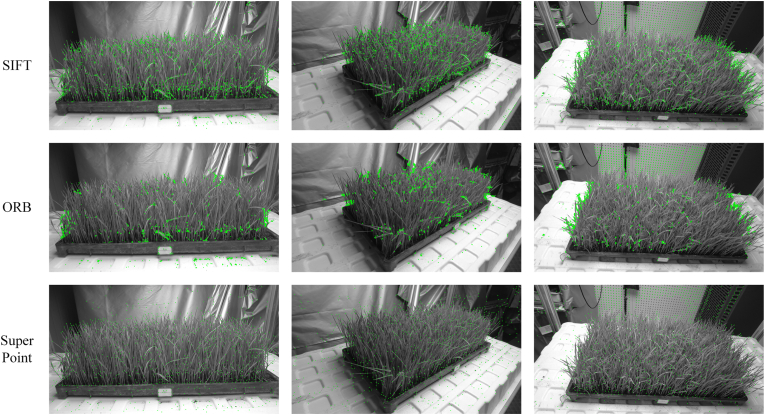
Feature point extraction comparison across different algorithms.

As shown in Fig. 4, the experimental results reveal that on the same rice seedling image dataset, SuperPoint extracts the fewest feature points, followed by ORB, while the SIFT extracts the most feature points. However, although the SIFT extracts the largest number of feature points, its robustness is poor, with significant numbers of redundant and low-quality feature points, especially in the edge regions of rice seedling leaves where many similar feature points exist, and it performs poorly in low-texture regions. In contrast, ORB, which relies on FAST corner detection, is significantly affected by the fine structures of the plants, resulting in highly clustered feature points and almost no responses in the low-texture regions of the rice. On the other hand, SuperPoint demonstrates a more uniform feature point distribution and can still extract discriminative feature points in low-texture regions, exhibiting stronger robustness and adaptability. Particularly in the edge and intersection regions of the rice leaves, the extracted feature points exhibit good spatial distribution characteristics. This finding indicates that although the SIFT and ORB have advantages in terms of the number of features, SuperPoint is better suited for conducting feature extraction in densely planted rice seedling scenarios because of its superior feature quality. The average feature extraction times required for the three algorithms are as follows: the SIFT takes 0.427 ​s, ORB is faster at 0.18 ​s, and SuperPoint takes the longest time at 0.526 ​s. With its relatively low computational complexity, the ORB algorithm performs optimally in terms of feature extraction speed, but its feature discriminability is insufficient, making it difficult to satisfy the requirements of the subsequent high-precision matching tasks. In contrast, although SuperPoint takes 23.1 ​% longer than the SIFT on average for feature extraction, its deep learning-based feature representation capabilities offer its significant advantages, allowing it to better adapt to complex texture changes and viewpoint transformations. Considering the strict feature quality requirements imposed 3D reconstruction tasks, this increase in time is within an acceptable range, making SuperPoint the superior choice.

#### 3.2.2 LightGlue feature matching evaluation

**Fig. 5:**
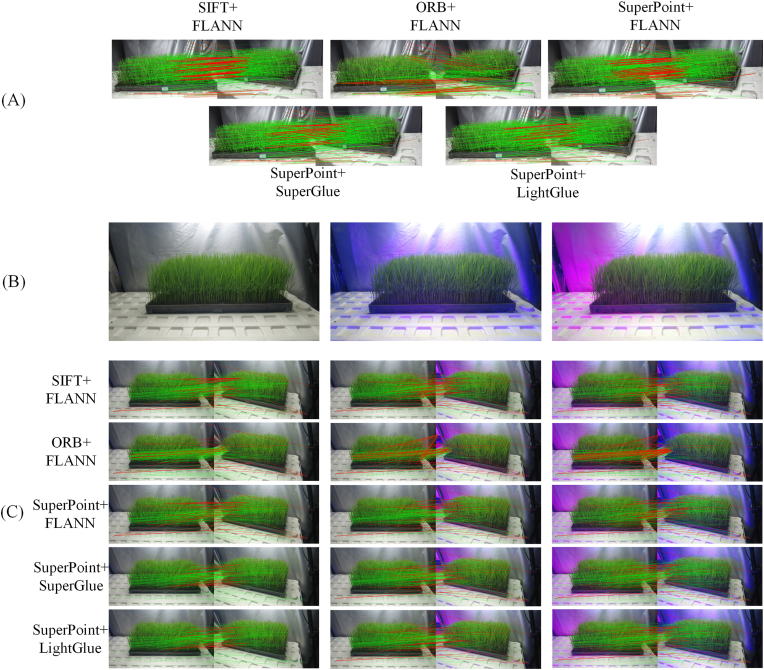
(A) Comparison among feature matching algorithms under normal lighting conditions. (B) Complex lighting variations in the same scene. (C) Comparison among feature matching algorithms under complex lighting conditions.

To comprehensively evaluate the feature matching performance of LightGlue on a multiview image dataset of rice seedlings, multiple comparative experiments are designed in this study, focusing on conducting quantitative analysis from two perspectives: the matching capabilities and computational efficiency of the tested methods. In addition, to further validate the matching performance of LightGlue in complex lighting environments, rice seedling scenes acquired under different lighting conditions are captured, as shown in Fig. 5(B). Owing to the diffuse reflection effect of the plant growth light, the light intensity changes in different regions of the image, which in turn affects the brightness and color distribution of the captured images. Therefore, the repeatability (Equation (16)), localization error (Equation (17)), and number of matching pairs are selected as evaluation metrics to comprehensively assess the matching performance of LightGlue in complex environments.

Rep (repeatability) refers to the proportion of feature points that are present in both images out of the total number of feature points. Suppose that image 1 contains N feature points and that image 2 contains M corresponding feature points. If the pixel distance between the feature points in image 1 after undergoing homographic transformation and the feature points in image 2 is smaller than the predefined threshold, the feature points are considered to exist in both images. The degree of repeatability is calculated using Equations (15), (16). In Equation (15), corr(*Χ*_*i*_) represents the number of repeatable feature points from image 1 that can be found in image 2, and corr(*Χ*_*j*_) represents the number of repeatable feature points from image 2 that can be found in image 1. ε is the predefined pixel distance threshold.

(15) $$\begin{array}{c} corr\left(X_{i}\right)=\left(min_{j\in \left\{1,\cdots N_{2}\right\}}\left\|X_{i}-{\hat{x}}_{j}\right\|\right)\leq \varepsilon \end{array}$$

(16) $$\begin{array}{c} Rep=\frac{1}{N+M}\left(\sum_{i}corr\left(x_{i}\right)+\sum_{j}\text{corr}\left(x_{j}\right)\right) \end{array}$$

The localization accuracy is calculated by comparing the corners detected by the algorithm with the real points in the image. The average minimum pixel distance between the detected corners and the true corners is calculated using Equation (17).

(17) $$\begin{array}{c} LE=\frac{1}{N}\sum_{i}\min_{j}\left\|x_{i}-{\hat{x}}_{j}\right\| \end{array}$$

The correctly matched feature point pairs detected by the algorithm are represented by green lines, while the incorrectly matched point pairs are represented by red lines. The experimental results indicate that ORB, which is limited by its feature extraction performance, tends to produce clustered feature points in overlapping rice leaf regions, leading to suboptimal cross-view feature matching quality. While the SIFT algorithm has an advantage in terms of the number of feature points, it generates numerous similar gradient descriptors in repetitive texture regions (e.g., the edges of tillering seedlings), resulting in significant redundancy and similar feature points that are difficult to match, along with many mismatched pairs, as shown in Fig. 5(A). SuperPoint ​+ ​FLANN relies solely on Euclidean distance-based descriptor matching, which is inefficient in densely overlapping rice leaf regions. In contrast, SuperPoint ​+ ​LightGlue and SuperPoint ​+ ​SuperGlue demonstrate superior matching performance, outperforming both the SIFT and ORB in terms of the number of matched pairs, with higher robustness. SuperPoint ​+ ​LightGlue effectively performs global feature matching through its transformer self-attention mechanism, reducing the number of mismatches and achieving excellent performance on the utilized dataset. SuperPoint ​+ ​SuperGlue employs graph neural networks (GNNs) to model the relationships between feature points and performs well on the dataset. However, LightGlue adopts a lightweight design, reducing the imposed computational load and optimizing the transformer computation process, making it more efficient than SuperGlue and achieving more efficient feature matching effects. LightGlue better satisfies the efficiency and robustness requirements of this study.

Under complex lighting conditions, as shown in Fig. 5(C), all the algorithms perform well when the lighting conditions change slightly. However, as the lighting changes become more significant, the ORB ​+ ​FLANN and SIFT ​+ ​ORB algorithms, which rely on traditional feature extraction and matching strategies, experience decreases in their numbers of matched pairs, along with noticeable increases in their numbers of mismatches. Although ORB experiences a smaller decrease in the number of matched pairs, most of the results are mismatches, while SuperPoint ​+ ​FLANN performs relatively stably. This is primarily because these traditional methods are more dependent on common local features, which are prone to distortion or deformation under significant lighting changes, leading to a decline in their matching quality and more mismatches. In contrast, SuperPoint ​+ ​FLANN does not rely on traditional local features, and despite having fewer matched points than the deep learning-based matching methods do, it remains relatively stable. On the other hand, by introducing a deep learning-based matching strategy, LightGlue can still effectively identify a large number of robust matches even under significant lighting changes. According to the data in Table 1, compared with SIFT ​+ ​FLANN, ORB ​+ ​FLANN, and SuperPoint ​+ ​FLANN, SuperPoint ​+ ​LightGlue provides feature point repeatability (Rep) improvements of 24.4 ​%, 16.2 ​%, and 7.2 ​%, respectively. The localization errors (LE) are reduced by 8.9 ​%, 28.4 ​%, and 9.3 ​%, respectively. The Rep and LE values of SuperPoint ​+ ​LightGlue are similar to those of SuperPoint ​+ ​SuperGlue. In complex lighting environments, the repeatability and localization error advantages of SuperPoint ​+ ​LightGlue demonstrate its ability to effectively mitigate the impact of lighting variations, consistently identify feature points, and provide more precision matching results, thereby satisfying the imposed robustness requirements.

**Table 1:** Performance comparison among different feature matching algorithms.

Metrics	SIFT ​+ ​FLANN	ORB ​+ ​FLANN	SuperPoint ​+ ​FLANN	SuperPoint+ SuperGlue	SuperPoint+ LightGlue
Average Number of Matches.	3956	2247	2927	4482	4664
Execution Time(min)	14.8	9.4	13.6	24.8	19.3
Rep	0.614	0.657	0.713	0.771	0.764
LE	1.118	1.424	1.124	1.034	1.019

### 3.3 Rice images background removals

In this study, the DeepLabv3+ semantic segmentation network is employed to remove the backgrounds of the rice images. To scientifically evaluate the background removal performance of the proposed method, all the pixels are categorized into four groups: true positives (TPs) are pixels that are correctly identified as foreground pixels (plants or seedling trays); false positives (FPs) are background pixels that are incorrectly identified as foreground pixels; true negatives (TNs) are pixels that are correctly identified as background pixels; and false negatives (FNs) are foreground pixels that are incorrectly identified as background pixels. The evaluation metrics used for the segmentation task include the intersection over union (IoU), precision, and recall.

(18) $$\begin{array}{c} IoU=\frac{TP}{TP+FP+FN} \end{array}$$

(19) $$\begin{array}{c} Presion=\frac{TP}{TP+FP} \end{array}$$

(20) $$\begin{array}{c} Recall=\frac{TP}{TP+FN} \end{array}$$

The DeepLabV3+ model achieves an accuracy of 0.98, a recall rate of 0.952, and an intersection over union (IoU) of 0.971. The experimental results demonstrate that DeepLabV3+ can accurately segment the plant regions in most rice seedling image samples, effectively removing background interference. However, the segmentation accuracy of the model is influenced by factors such as changes in the shooting angle, plant overlap, and complex backgrounds. As the shooting angle increases, the background complexity increases, and the degree of plant overlap becomes more prominent, leading to a decrease in the feature region recognition accuracy of the model. Specifically, under small-angle shooting conditions, the model can accurately separate the plant region. However, under larger angles or higher background complexity levels, the edge precision of the segmentation results decreases, causing minor mis-segmentations and missed segmentations. Overall, the model performs well and satisfies the requirements of the subsequent 3D reconstruction process.

To evaluate the segmentation performance and efficiency of DeepLabV3+ on rice seedling images, a comparative analysis is conducted with classic background segmentation algorithms, including K-means clustering and the watershed algorithm. A detailed comparison among the segmentation results is provided in the Supplementary Materials (refer to Fig. S1 for further details). Compared with the traditional methods, DeepLabV3+ demonstrates clear advantages in terms of its segmentation accuracy and robustness, especially when handling images captured from different viewpoints. It effectively isolates rice seedlings acquired from complex backgrounds and reduces the occurrence of mis-segmentations. During the dense reconstruction stage, the use of segmented images effectively reduces the number of irrelevant feature points caused by background interference, thereby improving the geometric accuracy and reconstruction consistency of the dense point cloud.

### 3.4 Point cloud denoising evaluation

**Fig. 6:**
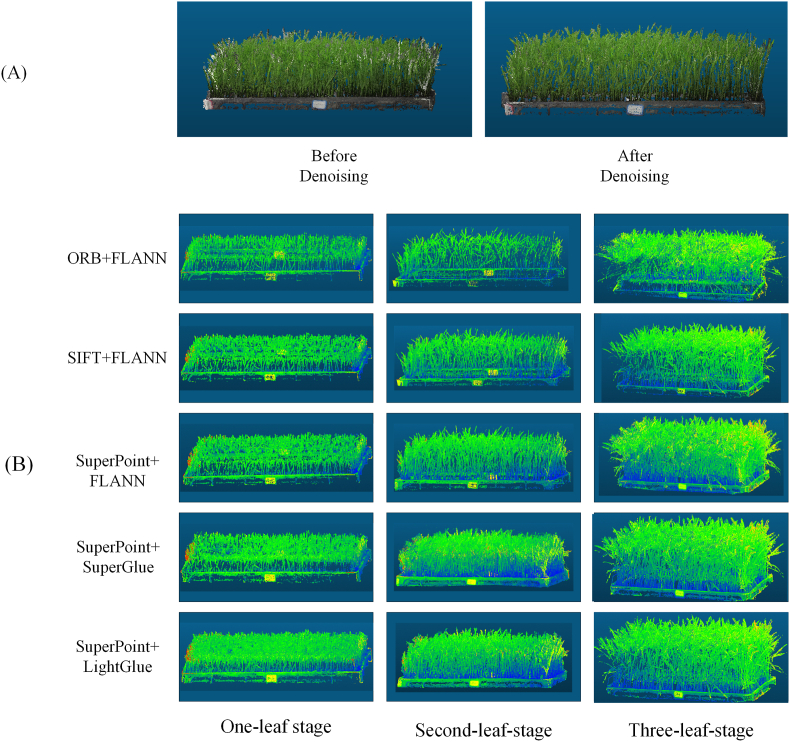
(A) Comparison among the point cloud denoising results. (B) Comparison among the reconstruction results obtained with different algorithms.

In the point cloud denoising experiment, after color filtering and statistical outlier removal (SOR) are applied, as shown in Fig. 6(A), the original point cloud without denoising contains a significant number of background noise points. These noise points are caused primarily by lighting reflections in the background regions, uneven textures, and incomplete image segmentation, leading to mismatched points. After processing the point cloud with the denoising algorithm, the number of outliers is significantly reduced, the density of the point cloud becomes more uniform, and the boundaries become clearer, resulting in a notable improvement in the quality of the point cloud data.

### 3.5 Evaluation of 3D reconstruction algorithms

#### 3.5.1 The accuracy of the SuperPoint ​+ ​LightGlue-SFM algorithm

To comprehensively evaluate the performance of the proposed multiview 3D reconstruction method, different reconstruction schemes are designed and compared in this study. On a unified experimental platform and dataset (images of rice seedlings obtained during the growth period), sparse reconstruction is implemented using COLMAP, with five different feature extraction and matching algorithms selected. Dense reconstruction is uniformly performed by using OpenMVS for the dense point cloud generation task. The reconstruction performance of the selected methods is thoroughly compared and analyzed using metrics such as point cloud completeness, reprojection error, the average trajectory length, and computational efficiency.

**Fig. 7:**
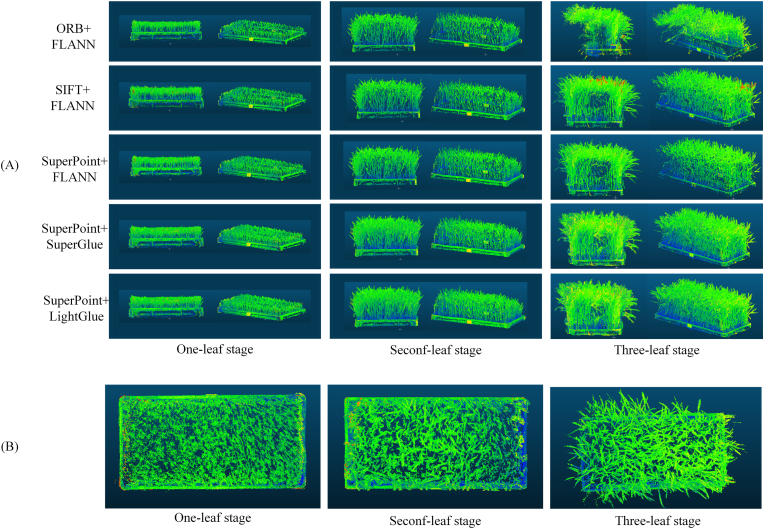
(A) Multiview comparison among the reconstruction results obtained from different algorithms. (B) Overhead view of different stages.

The point cloud reconstruction results obtained using different methods are shown in Fig. 6, Fig. 7. To enhance the visibility of the point cloud and display the details of different regions, thus making the morphologies of the rice seedlings more distinct and clearer, the RGB channels of the point cloud are synthesized using the formula Composite = (R ​+ ​G ​+ ​B)/3, resulting in a colored 3D visualization. The experimental results indicate that when the ORB method is used for 3D reconstruction purposes, the generated point cloud is incomplete, with noticeable gaps, especially in the complex areas of the rice seedling structure, such as the junctions between the stem and leaves. This missing data issue leads to reductions in both the completeness and accuracy of the reconstruction results. In contrast, the SIFT/SuperPoint ​+ ​FLANN method generates a more complete point cloud, but some loss of detail still occurs, and the reconstruction accuracy is insufficient, with the details not being as precise as those of the proposed method. The SuperPoint ​+ ​LightGlue and SuperPoint ​+ ​SuperGlue methods demonstrate the best reconstruction completeness in the 3D rice seedling reconstruction task, particularly in the stem region, where the point cloud is more complete and coherent. These methods are better at preserving details and complex structures, significantly improving their reconstruction quality.

**Fig. 8:**
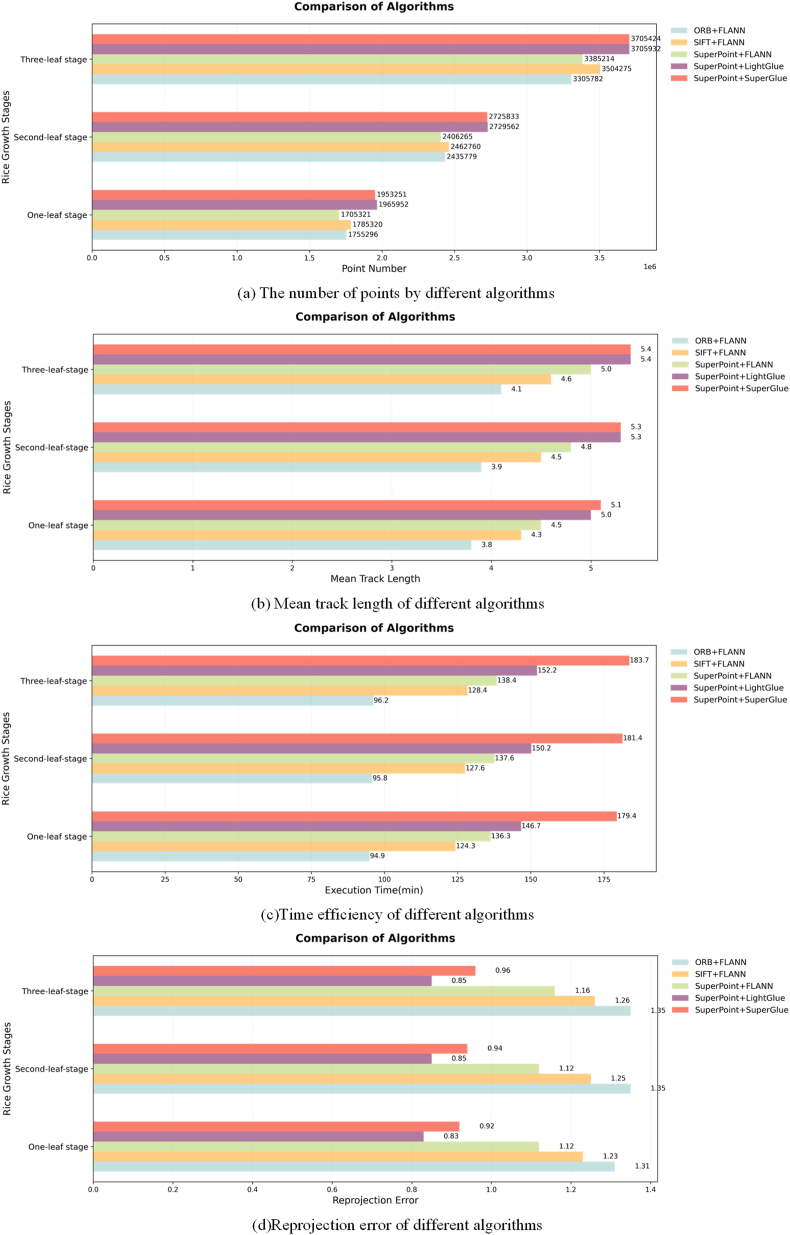
Comparison among the metrics produced by different algorithms at various plant growth stages.

As shown in Fig. 8, in this study, the reconstruction performances achieved by different algorithms during the one-leaf, two-leaf, and three-leaf stages of rice seedlings are systematically evaluated using core metrics such as the point cloud quantity and computational efficiency. The data in Fig. 8(a) indicate that compared with ORB ​+ ​FLANN, SIFT ​+ ​FLANN, and SuperPoint ​+ ​FLANN, SuperPoint ​+ ​LightGlue generates the greatest number of point clouds, with average performance improvements of 12.03 ​%, 8.91 ​%, and 12.7 ​%, respectively. On the same dataset and under the same experimental conditions, this algorithm achieves the longest average trajectory length, highlighting its ability to more stably and robustly track feature points across multiple views (Fig. 8(b)). Notably, as shown in Fig. 8(d), the reprojection error of SuperPoint ​+ ​LightGlue is lower than those of the other similar algorithms, with reductions of 36.9 ​%, 32.35 ​%, 25.57 ​%, and 10.27 ​% compared with the errors induced by ORB ​+ ​FLANN, SIFT ​+ ​FLANN, SuperPoint ​+ ​FLANN, and SuperPoint ​+ ​SuperGlue, respectively. These advantages contribute to improving the quality of the 3D reconstruction results, which is specifically manifested in the enhanced completeness of the plant morphologies.

The reconstruction times required for each sample at different stages are presented in Fig. 8(c). Compared with those of the traditional SIFT ​+ ​FLANN method, the computational times of SuperPoint ​+ ​LightGlue and SuperPoint ​+ ​SuperGlue increase by 18.06 ​% and 43.15 ​%, respectively. In densely planted rice seedling scenarios, owing to occlusion and interference caused by similar textures, the traditional methods that rely on local features are prone to mismatches. In contrast, the deep learning methods significantly improve their matching accuracy through global modeling and context awareness, albeit at the cost of increased computational resource consumption levels. However, compared with SuperGlue, which also uses a deep learning-based matcher, the SuperPoint ​+ ​LightGlue method saves 17.53 ​% of the total computational time. This is primarily due to introduction of a more lightweight transformer architecture and an adaptive key point selection mechanism by LightGlue, effectively reducing the number of redundant calculations. The proposed SuperPoint ​+ ​LightGlue-SFM method achieves the best overall performance in terms of the reprojection error, accuracy, and point cloud completeness metrics. This method provides higher reconstruction accuracy and superior point cloud quality for 3D reconstruction tasks involving densely planted rice seedlings, offering a more reliable three-dimensional data foundation for the subsequent phenotypic analysis. Although the SuperPoint ​+ ​LightGlue method requires more computational time than the traditional methods do, this is acceptable in practical applications. During the rice seedling cultivation process, phenotypic monitoring is typically conducted on a “daily” scale, with no real-time requirements, and the computational efficiency of the current method can satisfy the time scale demands of phenotype monitoring. Additionally, the traditional manual phenotype measurement method requires destructive sampling and involves tedious steps such as washing seedlings and arranging them on trays, leading to seedling losses and data biases.

#### 3.5.2 Phenotype measurement evaluation

**Fig. 9:**
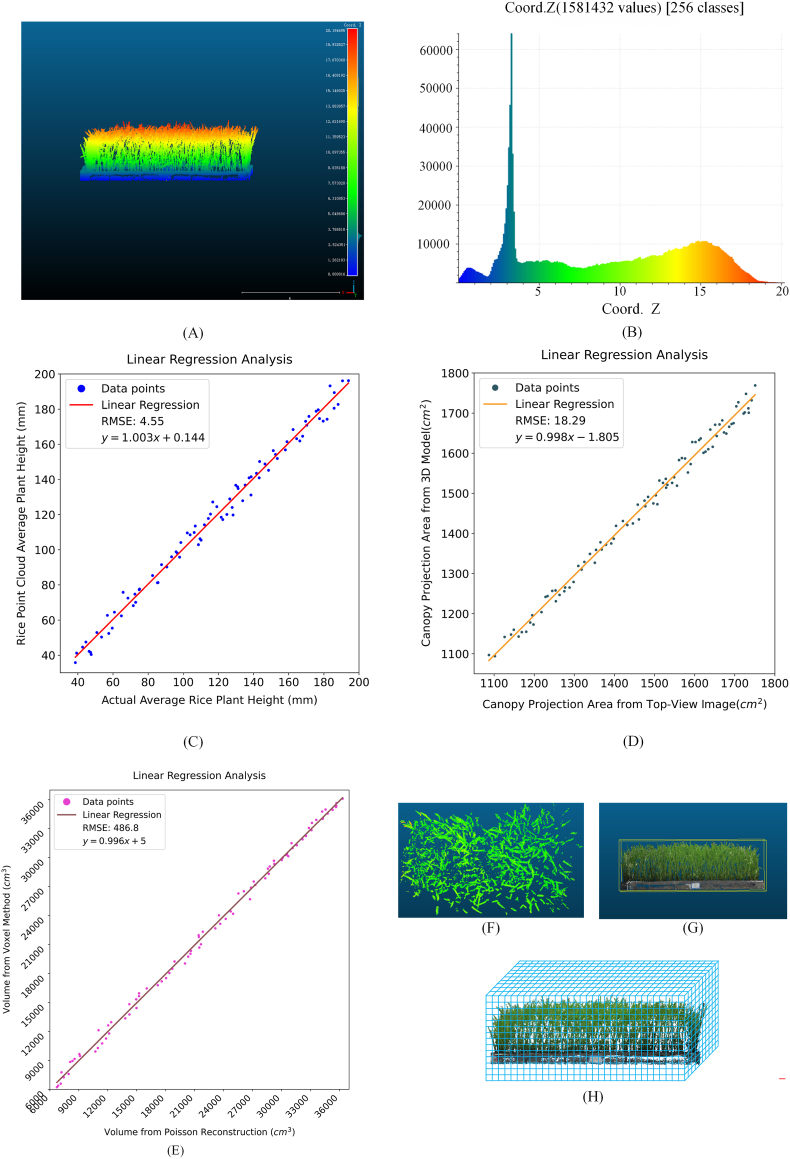
(A) Visualization of the point cloud elevation heights. (B) Height distribution histogram. (C) Regression analysis between the calculated and measured plant heights. (D) Regression analysis of the canopy projection areas derived from the 3D model and calculated from top-view images. (E) Regression analysis between the rice plant volumes of the voxel method and Poisson reconstruction. (F) Cross-section of the rice canopy point cloud in the three-leaf stage. (G) Schematic diagram of the bounding box-based volume calculation process. (H) Schematic diagram of the voxel-based volume calculation process.

Based on the cleaned point cloud data obtained after denoising and filtering, the average plant height of the rice seedlings is extracted as a key phenotypic parameter. Considering the dense planting conditions, directly using the maximum Z value as the plant height estimate is not feasible because the top point cloud is typically sparse and may contain noise or outliers (e.g., individual seedlings growing significantly taller than the overall height, whose highest points do not represent the overall height), which do not reflect the uniformity of the population growth process. Therefore, a robust statistical estimation method is employed in this study[40], with the mean of the top 5 ​% of the Z-coordinate points selected as the average plant height estimate. This approach provides a more scientific estimate of the overall average plant height of rice seedlings and reduces the impact of outliers on the height estimation results. The height information visualization and height distribution histogram are shown in Fig. 9(A) and (B), respectively.

On the basis of the manually measured data obtained from the data acquisition procedure described in Section 2.1.2, a linear regression analysis is conducted between the average height of the rice seedlings calculated from the point cloud and the actual measured values. As shown in Fig. 9(C), the average height of the rice seedlings calculated from the point cloud is strongly correlated with the manually measured average height, with a coefficient of determination *R*^2^ of 0.989, a root mean square error (RMSE) of 4.54 ​mm, and a relative root mean square error (rRMSE) of 3.88 ​%. The results indicate that the developed point cloud reconstruction method can accurately extract the height information of rice seedlings, validating the reliability and precision of the proposed method for the phenotypic measurement of seedling heights.

Owing to the severe structural overlap of rice seedling leaves under dense planting conditions, the internal leaf structure cannot be fully captured. Therefore, the traditional methods for measuring leaf areas based on fully expanded leaves are not applicable in this scenario. To address this issue, the projected canopy area is employed in this study as a surrogate indicator, and it is complemented by a cross-section canopy analysis. Specifically, the projected canopy area is calculated based on the orthographic projection of the three-dimensional model (projected vertically onto the horizontal plane along the Z-axis), which reflects the horizontal coverage of the canopy and its capacity for light interception. The top-view 3D models produced at various growth stages are shown in Fig. 7(B). In the early one-leaf stage, the projected area of the 3D model reaches approximately 0.11 ​m^2^, covering approximately 61.3 ​% of the standard seedling tray area, indicating that even in the early stage, the seedlings have already experienced a significant canopy coverage effect. In the early two-leaf stage, the projected canopy area increases to approximately 0.14 ​m^2^, and in the early three-leaf stage, the canopy continues to expand, with the projected area reaching approximately 0.16 ​m^2^, covering nearly 89 ​% of the tray area. To validate the accuracy of the projected canopy area calculated by the 3D model, top-view images of the rice seedlings are captured at various growth stages. Green pixels representing the seedling regions are extracted from each top-view image, counted, and then converted into the actual projected canopy area through scale calibration using a reference object (a standard seedling tray). A correlation analysis is performed to evaluate the consistency between the projected canopy areas obtained from the 3D models and those calculated from the top-view images. As shown in Fig. 9(D), the coefficient of determination *R*^2^ is 0.991, the root mean square error (RMSE) is 18.29 ​cm^2^, and the relative root mean square error (rRMSE) is 1.75 ​%, indicating that the canopy area measurement method based on 3D model projection is highly precise and reliable. In addition to the projected canopy area, the structural distribution of the seedlings in the cross-sectional layer provides a direct representation of the canopy status. Therefore, in this study, a representative height layer (Z ​= ​16.3 ​cm ​± ​0.5 ​cm) from the main canopy distribution area in the early three-leaf stage is selected for a cross-sectional point cloud analysis, as shown in Fig. 9(F). The results show that the point cloud in this layer is continuous and dense, exhibiting a bandlike expansion pattern, reflecting that the canopy of the rice seedlings has merged in the horizontal direction and no longer presents isolated growth patterns. Moreover, this layer exhibits good spatial coverage and structural closure effects. However, certain localized gaps remain in the canopy structure, possibly because of natural leaf tilting, growth posture changes, or occlusion. Additionally, during the one-leaf and two-leaf stages, the gaps within the canopy increase, and the canopy has not yet formed an effective horizontal continuous structure. Combining the results of the projected canopy area and the cross-sectional analysis, it can be concluded that, even though accurately measuring the leaf area is difficult, these structural surrogate parameters based on 3D point cloud models can still effectively characterize the canopy phenotype traits, reflecting the horizontal canopy coverage and the capacity for light interception, thus demonstrating promising applicability and practical value.

The volume of rice seedlings is among the key parameters for assessing the phenotypic characteristics of a population. The traditional bounding box volume calculation method, as shown in Fig. 9(G), typically relies on fitting the external boundary of point cloud data to derive an enclosing volume. However, the rice canopy is not completely compact, with certain gaps and irregularities. This results in overestimation, making the bounding box method often inaccurate in terms of reflecting the true volumes of rice seedlings. To address this issue, we adopt the voxel-based method for volume calculation purposes, as shown in Fig. 9(H). First, the point cloud data are voxelized with a predefined voxel size of 5 ​mm, dividing the point cloud into small cubic voxels. By counting the number of voxels containing the point cloud data and multiplying by the volume of each voxel, the overall volume can be estimated. As shown in Table 2, there are significant differences between the volume measurements of the two methods during the early leaf and third leaf stages. This is mainly due to the larger gaps between the seedlings in the early leaf stage, where the voxel method can more accurately adapt to the irregular canopy structure and effectively exclude the gaps. In contrast, the bounding box method tends to include these gaps as part of the volume, leading to overestimation. In the third leaf stage, as the canopy expands outward, the bounding box method includes the gaps between the curved leaves and outer stems of the canopy, which results in an overestimation of the volume. In the second leaf stage, the plant canopy expands, the gaps between plants are smaller, and the growth process is more uniform, resulting in minimal differences between the two methods. To further validate the accuracy of the voxel-based method for measuring the volumes of rice seedling populations, in this study, the volume values measured by Poisson surface reconstruction are selected as an indirect benchmark. This is because the true volumes of living seedlings cannot be directly measured (destructive measurements would damage the overall structures of the seedlings, affecting the experiment). First, the point cloud data are subjected to Poisson reconstruction (octree depth ​= ​10) to generate a closed, continuous mesh model with topological consistency. Afterward, on the basis of the Gauss divergence theorem[41], the closed volume is calculated by integrating the normal vectors of each triangular facet of the mesh, with the calculation performed using the Open3D library. A correlation analysis is performed between the seedling volumes measured by the voxel-based method and those obtained after performing Poisson reconstruction. The results (Fig. 9(E)) show an *R*^2^ of 0.984, an RMSE of 486.7 ​cm^3^, and an rRMSE of 2.18 ​%, indicating high consistency between the two methods and further confirming the accuracy of the voxel-based volume measurements. Notably, the voxel-based method offers higher computational efficiency, enabling rapid volume estimation processes to be performed through a simple 3D voxelization scheme without the need for complex surface reconstruction steps. In conclusion, by employing high-precision 3D reconstruction methods, 3D point cloud models of rice seedlings at different growth stages are constructed, enabling the phenotypic features of rice seedlings to be more accurately quantified and providing reliable data support for subsequent phenotype analyses and research.

**Table 2:** Comparison among the volume estimates produced for rice seedlings at different growth stages using bounding box and voxel methods.

Growth stage	Algorithm	Minimum volume(m^3^)	Maximum volume(m^3^)	Standard deviation (m^3^)	Variance (m^6^)	Mean (m^3^)
One-leaf stage	bounding box	6.95 ​× ​10^−3^	9.52 ​× ​10^−3^	9.79 ​× ​10^−4^	9.57 ​× ​10^−7^	7.42 ​× ​10^−3^
	voxel	6.32 ​× ​10^−3^	9.15 ​× ​10^−3^	9.24 ​× ​10^−4^	8.53 ​× ​10^−7^	7.39 ​× ​10^−3^
Two-leaf stage	bounding box	9.63 ​× ​10^−3^	2.12 ​× ​10^−2^	7.40 ​× ​10^−4^	5.48 ​× ​10^−7^	1.64 ​× ​10^−2^
	voxel	9.46 ​× ​10^−3^	1.92 ​× ​10^−2^	7.14 ​× ​10^−4^	5.10 ​× ​10^−7^	1.57 ​× ​10^−2^
Three-leaf stage	bounding box	1.75 ​× ​10^−2^	3.97 ​× ​10^−2^	2.82 ​× ​10^−3^	7.95 ​× ​10^−6^	3.13 ​× ​10^−2^
	voxel	1.49 ​× ​10^−2^	3.61 ​× ​10^−2^	1.33 ​× ​10^−3^	1.77 ​× ​10^−6^	2.87 ​× ​10^−2^

## 4 Discussion

During the multistage 3D rice plant reconstruction process, the key factors that affect reconstruction accuracy, including the camera angle, image capture quality, and image background, are carefully considered. To address the potential reconstruction errors caused by these factors, a “camera-to-rice seedlings” platform is constructed, and the 3D reconstruction workflow is optimized.

### 4.1 Discussion on the impact of the 3D reconstruction platform on reconstruction

During the image data acquisition process, the traditional rotating platform methods involve capturing images by rotating a platform along with the plant seedlings. While this approach conveniently obtains multiview image data, unavoidable vibrations and mechanical errors induced during platform rotation often cause seedling vibrations, especially at the leaf tips[28]. These vibrations introduce additional noise, thereby affecting the image clarity level and the subsequent 3D reconstruction accuracy [42]. Researchers typically employ improved 3D reconstruction platforms to address this issue [43]. utilized a customized mechanical structure with 10 cameras fixed at different angles on an arc-shaped bracket, which they combined with structured light to achieve multiview 3D plant reconstruction [44]. employed a custom circular rail system to control a camera, centering on the target plant to capture multiview images. However, these platforms are often costly.

To address the nonrigid plant seedling vibrations caused by the traditional rotating platform methods, a “camera-to-rice seedling” platform is constructed in this study, replacing plant rotation with camera movement and effectively reducing the vibration issues caused by platform rotation. Additionally, compared with the traditional 3D reconstruction platforms, this platform incorporates 30^o^ and 60^o^ shooting capabilities, enabling the acquisition of image data from more perspectives and enhancing the coverage and information content of the image. A significant correlation is present between the acquisition angle and the resulting reconstruction accuracy [45]. quantified the impacts of different ROV camera angles on biological community assessments through a 3D simulation and reported that camera angles significantly affect species with relatively high 3D structures and that camera angle changes can lead to the loss of key structural information. Similarly, for densely planted rice seedlings, the angle influences the ability to capture the morphological features of plants. Low-angle (0^o^ and 30^o^) images effectively reconstruct the basal structures of stems and the spatial distributions of leaves, whereas high-angle (60°) images optimize the process of reconstructing geometric details at the canopy top [24]. The absence of high-angle image data can result in insufficient geometric details of the target plant canopy and reduced overall structural integrity. To balance point cloud completeness and computational efficiency, during the early growth stages of one- and two-leaf rice seedlings, only 0^o^ and 30^o^ images need to be captured to reduce the degree of data redundancy and achieve improved processing efficiency. Additionally, considering the relatively simple traits of rice in its early stages, the number of images can be appropriately reduced to further increase the overall speed of data processing.

### 4.2 Discussion on denoising strategies

Point cloud noise typically originates from multiple factors, including background noise, lighting variations, and matching errors. Background noise arises from nontarget objects, leading to the inclusion of redundant data in the reconstruction results [46]. proposed a method called nontarget background removal and depth fusion (NBR-DF), which reduces noise in depth images by eliminating their backgrounds and extracting target objects; thus, noise in the depth images is reduced, thereby improving the resulting detection accuracy. In this study, a two-stage reconstruction strategy for static rice seedlings is adopted. The first stage involves performing sparse reconstruction based on the original images, whereas the second stage consists of using DeepLabV3+ for conducting background segmentation on the images. By combining sparse reconstruction results with segmented images, depth information fusion generates point clouds containing only the target objects. With their rich background information, the original images provide a sufficient source of information for key point detection and matching [35]. Directly using the background-removed images for sparse reconstruction purposes is found to require multiple optimization iterations or even result in reconstruction failures during the experiments, thereby increasing the computational time. The use of segmented images in the dense stage avoids the calculation of numerous irrelevant points, saving computational resources and providing improved accuracy. Despite the two-stage reconstruction strategy, the model still contains significant noise, primarily because of the residual errors induced in the background of the segmented image and the effects of light reflections. During the initial experiments, the desktop of the platform is not covered with photographic light-absorbing cloth, causing reflections from the desktop because of the supplemental lighting and Philips lighting in the artificial climate chamber. This results in a large number of white noise points near the edges of the plant point cloud leaves. To address these issues, HSV color space-based filtering and statistical outlier detection methods are employed during the point cloud postprocessing stage. Researchers (D. [24]) have used color filtering to effectively remove black noise at leaf edges and white noise between stems, whereas [47] applied statistical filtering to eliminate the outliers and noise around soybean canopies. The combination of color filtering and statistical outlier detection can effectively remove noise around rice leaves. Additionally, we recommend covering the desktop with photographic light-absorbing cloth during shooting to fundamentally reduce the interference caused by light reflections with respect to the reconstruction quality.

### 4.3 Discussion on reconstruction algorithms

To address challenges such as the complex texture variations and high structural similarity among plants in densely planted rice scenarios, an improved 3D reconstruction method using SuperPoint ​+ ​LightGlue is employed in this study. The traditional handcrafted feature extraction methods often struggle to accurately extract and match the key points in scenes with complex textures and similar structures, leading to errors or low precision levels during reconstruction[48]. Researchers typically adopt deep learning methods to improve the effectiveness of the feature extraction and matching processes. For example [49], introduced D2-Net, which is a novel local feature detection and description method that simultaneously performs detection and description on local features, providing more stable matching capabilities in scenes with lighting variations [50]. presented the SuperGlue neural network model, which uses graph neural networks to learn to match features; this approach, replaces the traditional nearest-neighbor matching methods and provides enhanced matching stability and accuracy. To handle densely distributed and structurally similar rice seedlings, the SuperPoint ​+ ​LightGlue 3D reconstruction method is adopted in this study, leveraging the hierarchical feature representation advantages of deep learning to surpass the performance limits of traditional handcrafted features. Through a multiscale feature pyramid, it effectively enhances the local contrast level, achieving improved reconstruction accuracy in low-texture regions [51]. The local contrast levels is enhanced in low-texture regions at the stem base. During the matching stage, adaptive feature filtering reduces the number of ambiguous matches in densely featured areas, enabling point cloud models for rice seedlings to be more accurately and completely constructed in different growth stages.

During the phenotypic analysis phase, to prevent uneven growth from impacting the results, we do not measure the small portion of the rice plants in the seedling trays that exhibit significantly delayed growth. Only plants with consistent growth states and representative characteristics are measured. The phenotypic measurements derived from the 3D models are validated against reference measurements for each parameter. For the rice plant height, the correlation yields an *R*^2^ of 0.989, an RMSE of 4.54 ​mm, and an rRMSE of 3.88 ​%. For the projected canopy area, *R*^2^ ​= ​0.991, RMSE ​= ​18.29 ​cm^2^, and rRMSE ​= ​1.75 ​%. For the rice plant volume, *R*^2^ ​= ​0.984, RMSE ​= ​486.7 ​cm^3^, and rRMSE ​= ​2.18 ​%. All the parameters demonstrate high accuracy. With rRMSE values ​≤ ​10 ​%, the results indicate that the phenotypic measurements obtained in this study hold significant reference value for plant phenotyping[44]. Additionally, the use of 3D measurement technology for extracting phenotypic parameters enables nondestructive and high-throughput phenotypic data collection processes [52]. successfully distinguished banana volume traits under drought stress using 3D point cloud models [53]. obtained multiple phenotypic parameters from skeleton and reconstructed point clouds on the basis of 3D models, demonstrating the potential of their method for predicting dry and wet biomass. These studies further validate the application potential of this method for screening rice varieties with superior growth traits. In addition, barley possesses a similar cultivation pattern to that of rice, where seedlings are also densely grown in standard seedling trays during the early growth stage. The structural characteristics of barley seedlings are highly similar to those of rice seedlings. Therefore, the 3D reconstruction and phenotypic measurement method proposed in this study is also feasible for applications involving barley crops.

Although the proposed 3D reconstruction method demonstrates significant advantages in experiments, it still has several limitations. First, the method relies on deep learning models during its feature matching stage, which increases its computational complexity relative to that of the traditional matching methods. This is particularly evident when processing high-resolution images, which require hardware devices with higher computational capabilities, somewhat limiting its practical applicability in resource-constrained environments. Second, the experimental data are primarily derived from densely planted rice seedling scenarios under controlled conditions, with relatively limited dataset coverage that does not include other crop types or complex field environments. Therefore, the generalizability and adaptability of the proposed method to a wider range of plant species and natural environments require further validation.

## 5 Conclusion

A multiview image-based “camera-to-rice seedlings” 3D reconstruction platform is proposed to generate a highly precise point cloud model. The complete workflow includes five core steps: image acquisition, image segmentation, 3D reconstruction, point cloud denoising, and calibration. A multiview image acquisition strategy combined with a customized shooting device ensures comprehensive coverage of the target rice seedlings. DeepLabV3+ enables the accurate segmentation of plant foreground images. The adopted SuperPoint ​+ ​LightGlue-SFM 3D reconstruction method better preserves plant details in complex environments, enhancing the accuracy and completeness of point clouds. Additionally, through a two-stage reconstruction strategy combined with HSV color filtering and statistical outlier removal, noise can be effectively eliminated. Furthermore, when prior information derived from the seedling tray is used, the scale restoration method accurately recovers the sizes of the point clouds of the rice plants. The experimental results demonstrate that high measurement accuracy is achieved across all the phenotypic parameters: the plant height has an *R*^2^ of 0.989, with an RMSE of 4.54 ​mm; the canopy projected area has an *R*^2^ of 0.991, with an RMSE of 18.29 ​cm^2^; and the rice population volume has an *R*^2^ of 0.984, with an RMSE of 486.7 ​cm^3^.

The proposed 3D reconstruction method can be used for growth monitoring and key phenotypic parameter extraction tasks involving rice seedlings. However, compared with the traditional methods, this approach requires more computational resources, and the overall processing time needed for the multiview data acquisition, feature matching, and 3D reconstruction steps is longer. In the future, lightweight deep learning models will be further optimized, including by improving upon the image overlap situation (reducing the number of redundant images to lower the computational load) and implementing image subsampling strategies (balancing the resolution and speed of the model), to enhance the reconstruction efficiency of the method and further improve its practicality and scalability. Moreover, by integrating automated solutions, the entire workflow—from data acquisition, image transmission, preprocessing, and feature matching to 3D reconstruction and phenotypic parameter extraction—will be automated, establishing an efficient end-to-end reconstruction pipeline.

## CRediT authorship contribution statement

Zhigang Zhang: Conceptualization, Formal analysis, Methodology, Data curation, Writing - original draft. Liwei Wang: Formal Analysis, Investigation, Data Curation, Writing - review & editing. Weiqi Ren: Data curation, Formal analysis, Methodology. Shoutian Dong: Acquisition of data, Validation, Data curation. Shaowen Liu: Data curation, Validation, Resources. Haoran Xu: Data curation, Validation, Resources. Yubo Yang: Data curation, Validation. Rui Gao: Funding acquisition, Project administration, Resources, Supervision. Zhongbin Su: Funding acquisition, Investigation, Project administration, Resources.

## Declaration of interests

The authors declare that they have no known competing financial interests or personal relationships that could have appeared to influence the work reported in this paper.

## Data Availability

The data collected and/or analyzed during this study can be obtained from the corresponding author upon reasonable request. The code used in this study is available at https://github.com/Terrywewee/3D-reconstruction-of-densely-planted-rice-seedlings---superpoint-lightglue.git.
