# Supplementary material for: Three-dimensional reconstruction of densely planted rice seedlings based on MultiView images
Source: Plant Phenomics. 2025 Sep 30;7(4):100122. doi: 10.1016/j.plaphe.2025.100122 (PMC13109332; doi:10.1016/j.plaphe.2025.100122)
Supplement: Multimedia component 1 [file mmc1.docx]

# **Supplementary Materials**

**Table S1.** Detailed L9 orthogonal design for the experimental treatments.

| Experimental Group | Light quality  (Factor A) | Light quantity (Factor B µmol·m^-2^·s^-1^) | Photoperiod  (Factor C, h) |
| --- | --- | --- | --- |
| 1 | R: B=0.7 | 270 | 15 |
| 2 | R: B=0.7 | 340 | 12 |
| 3 | R: B=0.7 | 510 | 8 |
| 4 | R: B:FR=62.5:25:12.5 | 270 | 12 |
| 5 | R: B:FR=62.5:25:12.5 | 340 | 8 |
| 6 | R: B:FR=62.5:25:12.5 | 510 | 15 |
| 7 | W | 270 | 8 |
| 8 | W | 340 | 15 |
| 9 | W | 510 | 12 |


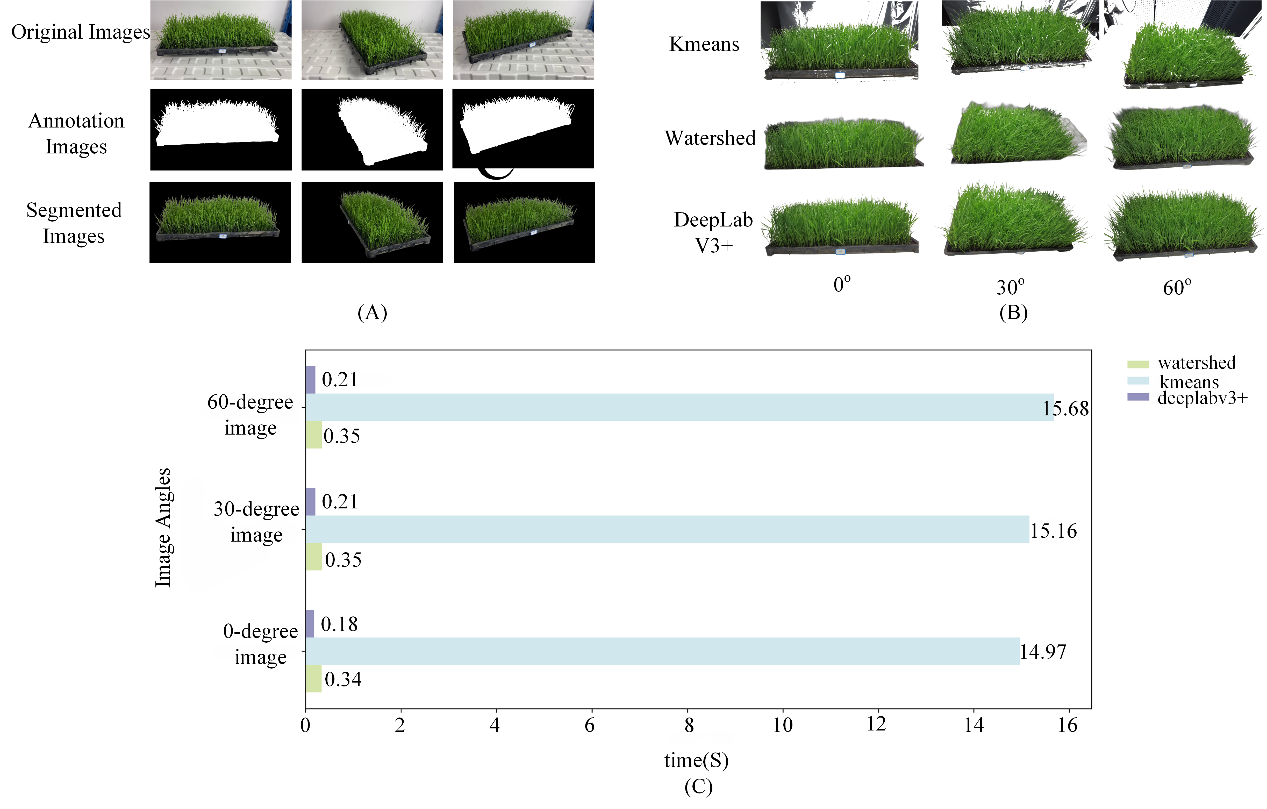


**Figure S1.** (A) Annotation and segmentation results obtained for the rice seedlings. (B) Comparison among the segmentation results produced by different algorithms across various shooting angles. (C) Comparison among the segmentation execution times required across different shooting angles.
